# Blood culture bottle shortage mitigation efforts: analysis of impact on ordering and patient impact

**DOI:** 10.1017/ash.2024.474

**Published:** 2025-01-09

**Authors:** Christopher D. Doern, Melissa Whitman, Michelle Doll, Suzanne Lavoie, David Friedel, Gonzalo Bearman, Jeffrey Kim, Heather Masters, Susan Roseff, Jim Willis, Roxanne Mercer, Aaron Hill, Ramana Feeser, Harinder Dhindsa, Frank Petruzella, Anne Jackson, Michael Vitto, Josh Plauny, Alexandra Bryson

**Affiliations:** 1 Department of Pathology, Virginia Commonwealth University School of Medicine, Richmond, VA, USA; 2 Department of Emergency Medicine, Virginia Commonwealth University School of Medicine, Richmond, VA, USA; 3 Department of Internal Medicine, Virginia Commonwealth University School of Medicine, Richmond, VA, USA; 4 Department of Pediatrics, Virginia Commonwealth University School of Medicine, Richmond, VA, USA; 5 Department of Family Medicine and Population Health, Virginia Commonwealth University School of Medicine, Richmond, VA, USA; 6 Virginia Commonwealth University Health System, Richmond, VA, USA

## Abstract

**Objective design:**

In June of 2024, Becton Dickinson experienced a blood culture bottle shortage for their BACTEC system, forcing health systems to reduce usage or risk exhausting their supply. Virginia Commonwealth University Health System (VCUHS) in Richmond, VA decided that it was necessary to implement austerity measures to preserve the blood culture bottle supply.

**Setting:**

VCUHS includes a main campus in Richmond, VA as well as two affiliate hospitals in South Hill, VA (Community Memorial Hospital (CMH)) and Tappahannock Hospital in Tappahannock, VA. It also includes a free-standing Emergency Department in New Kent, VA.

**Patients:**

Blood cultures from both pediatric and adult patients were included in this study.

**Interventions:**

VCUHS intervened to decrease blood culture utilization across the entire health system. Interventions included communication of blood culture guidance as well as an electronic health record order designed to guide providers and discourage wasteful ordering.

**Results:**

Post-implementation analyses showed that interventions reduced overall usage by 35.6% (*P* < .0001) and by greater than 40% in the Emergency Departments. The impact of these changes in utilization on positivity were analyzed, and it was found that the overall positivity rate increased post-intervention from 8.8% to 12.1% (*P* = .0115) and in the ED specifically from 10.2% to 19.5% (*P* < .0001).

**Conclusions:**

These findings strongly suggest that some basic stewardship interventions can significantly change blood culture practice in a manner that minimizes the impact on patient care.

## Introduction

Recently, Becton Dickinson experienced a blood culture bottle shortage for their BACTEC system creating a significant issue for those health systems which use their product. In the United States, there are only two major manufacturers of blood culture systems and while data demonstrating the market share is not available, it is expected that a sizable percentage of healthcare systems will be experiencing a blood culture shortage for the spring and fall of 2024. To preserve the ability to perform blood cultures, health systems are implementing diagnostic stewardship tactics to reduce blood culture orders.

This manuscript describes the measures taken by a large academic health system to control blood culture utilization and provides an analysis of those stewardship efforts in terms of their impact on utilization and patient care. The primary interventions utilized education and messaging as well as some specific guidance to providers to differentiate low-risk and high-risk patient populations for bacteremia. These measures and guidance documents were developed based on the existing blood culture stewardship guidance.^1,2^ It is hoped that this manuscript’s findings will prove useful to other institutions looking to control blood culture utilization, preserve their supply and help them understand the consequences of these measures.

## Materials and methods

Patient population: This study was conducted at Virginia Commonwealth University Health System (VCUHS) in Richmond VA. VCUHS contains VCU Medical Center (VCUMC) and Children’s Hospital of Richmond, both of which are urban, academic medical centers which care for both pediatric and adult patient populations. Included within the VCUHS are two community-based affiliates, Tappahannock Medical Center (Tapp) in Tappahannock, VA, and Community Memorial Hospital (CMH) in South Hill, VA. VCUHS also has a free-standing Emergency Department in New Kent, VA. All blood cultures from all VCUHS patients collected between June 1, 2022, and July 21, 2024, were analyzed.

Blood Culture Practices: Across VCUHS, providers are encouraged to collect two blood culture sets, each set including 10 ml aerobic and a 10 ml anaerobic bottle. In pediatrics, weight-based guidelines are utilized to promote drawing the maximum safe volume from the child.^3^ The Becton Dickinson (BD) Bactec blood culture system (Sparks, MD) is used throughout VCUHS. Adult bottles are used for all patients, including children.

Microbiology: The VCUHS main campus (Richmond, VA), Tappahannock, and CMH laboratories all incubate blood cultures on site, while the New Kent ED sends blood cultures to the main campus for incubation. Upon turning positive, each facility will perform a Gram stain, but CMH and Tappahannock then send their positive bottles to the VCUHS core Microbiology laboratory for subculture, BioMerieux Biofire blood culture identification PCR (BCID) (Marcy l’Etoile, France), organism identification, and susceptibility testing if indicated. Culture-based identification was performed using the Bruker Daltonics (Billerica, MA) Biotyper matrix assisted laser desorption ionization time-of-flight mass spectrometer (MALDI-TOF MS).

Analysis: All blood cultures collected between June 1, 2022, and July 21, 2024, were included in this analysis. Positive blood cultures were defined by any bottle (aerobic, anaerobic, or both) that was positive from a single source. Contamination was defined as any probable contaminant organisms (ie coagulase negative staphylococci, *Corynebacterium* spp., *Bacillus* spp. (non-anthracis), etc.…) that was only positive from a single culture. Clinically relevant positivity rates over a given period were calculated by subtracting each month’s contamination rate from the overall positivity rate.

**Figure 1.**
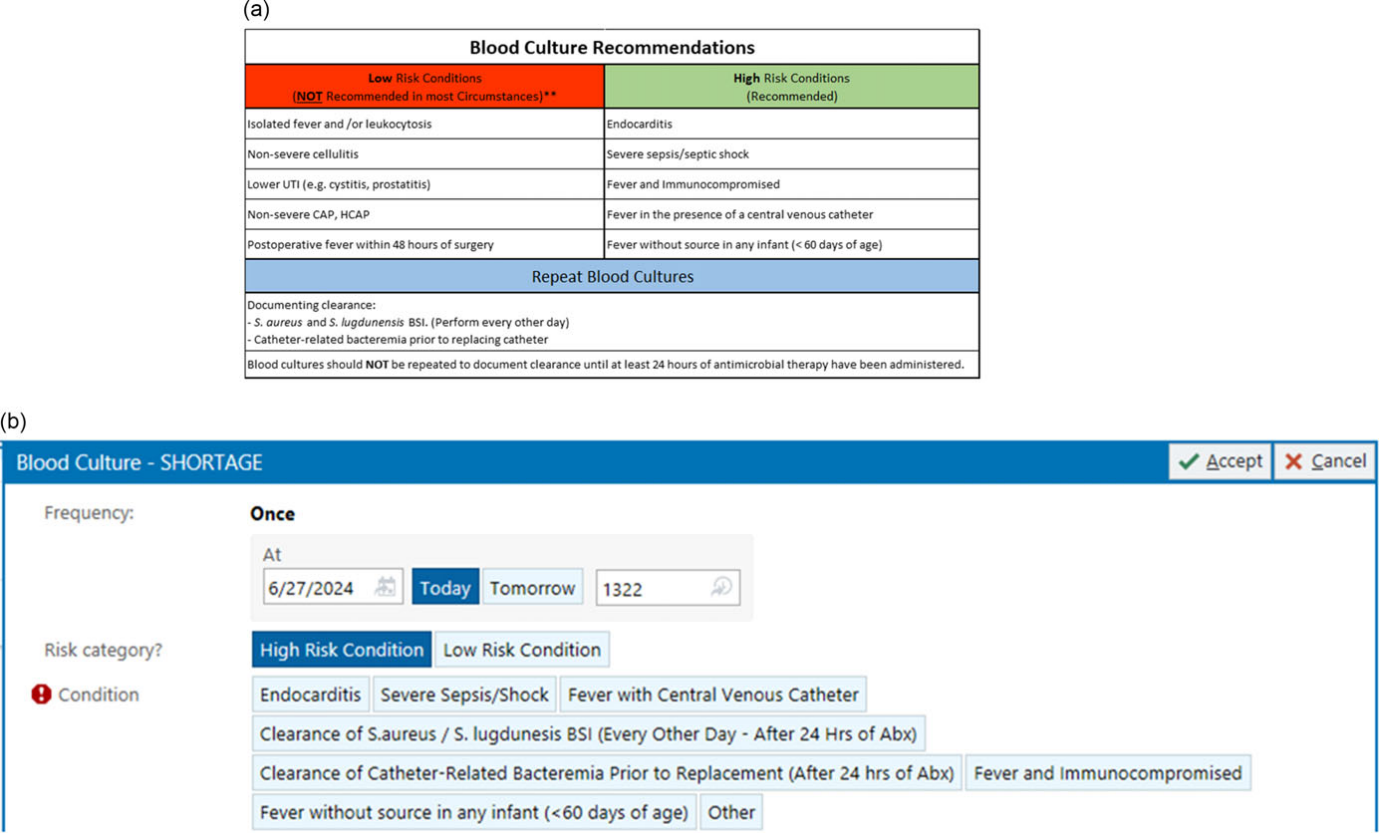
(a) Guidance communicated to the health system. (b) EPIC Best Practice Alert (BPA) which was implemented for blood culture orders.

Blood culture austerity measures: To encourage judicious use of blood culture and preserve the limited supply of blood culture bottles, VCUHS deployed multiple system-wide interventions. Guidance documents were developed by soliciting VCUHS provider input as well as a review of the literature.^1,2^ Passive interventions included mass messaging (via email) of guidance designed to help providers stratify the risk of bacteremia (Figure 1a). In addition, upon logging into the electronic medical record (EMR), providers were greeted with a brief message alerting them to the blood culture bottle shortage and asking that blood cultures be ordered judiciously (not shown). Hardcopy messaging about the shortage was also prominently displayed at each unit’s blood culture supply station. Targeted messaging was conducted in high use areas such as the intensive care units (ICU) and the Emergency Department (ED). July marks the beginning of the academic year when new resident physicians begin participating in patient care. Therefore, trainees were also targeted with messaging.

The primary active intervention was replacement of an existing blood culture order with a new order that forced providers to indicate whether the patient was at high- or low-risk of bacteremia. In this order, pre-populated high-risk conditions were presented as selectable options (Figure 1b). Low-risk conditions required free-text entry. In addition, all blood culture bottles were removed from the environment to obtain an accurate inventory. Once counted, reduced supplies were redistributed based on projected reductions in weekly usage. Units requiring more blood culture bottles would have to request additional supply.

This work met institutional criteria for quality improvement and did not meet HHS definitions of human subject’s research.

Summary statistics were calculated for blood culture volumes by day and by month in pre- and post- intervention time periods. The *T*-test was used to compare overall culturing rates (ie number of cultures per location per unit time), and the *Z*-test was used to compare culture positivity rates. Chi-square tests were used to compare the proportion of patients with single blood cultures during the austerity measures versus prior summer months (June–July).

## Results

### Changes in blood culture test volume

**Figure 2.**
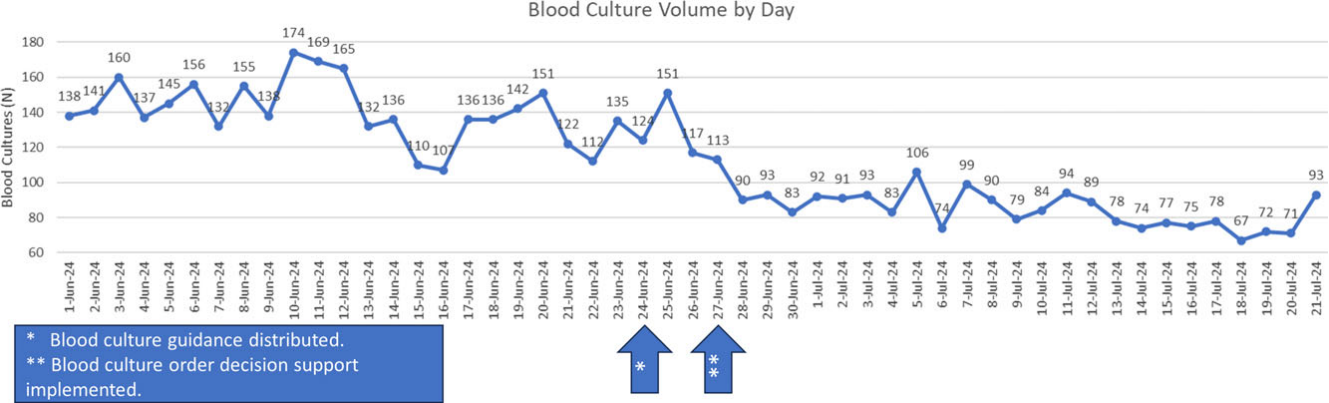
Daily blood culture order volume for immediate pre-intervention period (June 1–23^rd,^ 2024) and post-intervention period (June 24^th^ – July 21^st^, 2024).

**Figure 3.**
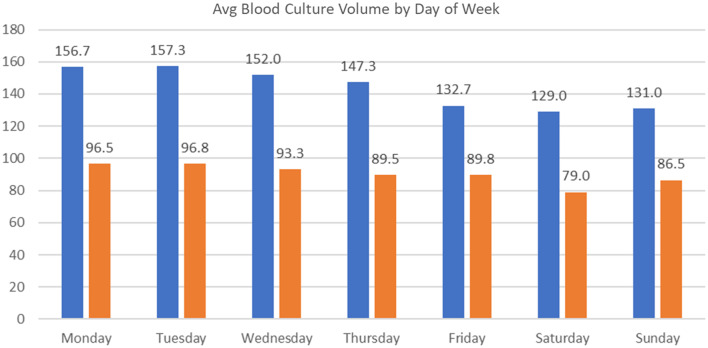
Pre- and post-intervention period blood culture ordering practices stratified by day of the week.

System wide messaging was distributed on June 24th and 25th, and the EPIC orderable intervention was introduced on June 27th, 2024 (Figure 1). We compared test volume to several different historical periods at VCUHS and assessed whether these interventions had an impact on blood culture ordering. For the purposes of this analysis, we will consider the austerity measures to have been implemented on June 24th, 2024. Between June 1st and June 23rd, 2024, VCUHS averaged 140.4 (SD 17.8) blood cultures per day. Between June 24th and July 21st, the health system averaged 90.4 (SD 18.4) blood cultures per day, a 35.6% reduction, *P* < .0001 (Figure 2). In addition, blood culture ordering varies by day of the week with Monday through Wednesday yielding higher volumes, averaging 155.3 (SD 15.5) cultures per day. Thursday through Sunday yield lower volumes, averaging 134.3 (SD 15.8) cultures per day. This trend continued in the post-intervention period with 95.5 (SD 23.9) and 86.2 (SD 13.4) cultures per day, in the Monday–Wednesday and Thursday–Sunday periods, respectively (Figure 3).

The post-intervention period was also compared to previous years. In June and July 2022 and 2023, the average blood culture volumes were 124.7 and 129.1 cultures per day, respectively. The average over the 2022 and 2023 (full academic year July–June) was 123.2 and 135.3 cultures per day, respectively. Regardless of the comparator period, the daily, post-intervention, blood culture volumes (89.6 cultures per day) were reduced.

The average daily volume was 111 cultures in the first week following intervention (June 24^th^, 2024). Volume continued to decrease in each successive week, with 90, 84, and 76 cultures per day ordered in the weeks of July 1st, 8th, and 15th, respectively. The 76 cultures per day ordered the week of July 15^th^, represent a 45.9% reduction (compared to 140.4 culture/day average – June 1 – 23, 2024).

Analysis of ED specific blood culture practices showed significantly decreased ordering. The main campus ED, the CMH ED, and the Tapp ED reduced ordering by 42%, 50.3%, and 49.5%, respectively when compared to averages for each location between June 1 and 23, 2024 (Table 1). Other higher volume areas of the health system were analyzed, including bone marrow transplant, medical respiratory intensive care unit (MRICU), and surgical trauma (STICU). All units saw a decrease in order volume that ranged from 15.8% to 47.2% reduction, with the changes in higher volume units being statistically significant (Table 1). The only unit which did not observe a decrease in usage was the Pediatric Emergency Department (CTWR ED).

**Table 1.** Unit specific changes in blood culture order volume

	Unit									
	Main ED	CMH ED	Tapp ED	CTWR ED	Bone Marrow Transplant	MRICU	Acute Care Oncology	STICU	MICU	Total
Preintervention * Average/ day	37.4	8.7	7.5	3.0	3.8	7.9	3.2	4.0	4.7	140.4
Postintervention * Average/day	22.1	4.4	4.0	3.0	3.0	5.5	2.3	3.6	2.6	90.4
% Change	−40.8%	−48.8%	−46.8%	0.0%	−20.7%	−30.1%	−30.1%	−9.7%	−45.0%	−35.6%
*P*-value	<.0001	.003	.0016	.8801	.4251	.1121	.1121	.5508	.0056	<.0001

* Preintervention period – June 1–23, 2024, Postintervention period – June 24 – July 21, 2024. CTWR – Children’s Tower ED, MRICU – Medical Respiratory ICU, STICU – Surgical Trauma ICU, MICU – Medical ICU.

**Table 2.** Proportion of patients with only a single set of blood cultures sent

Year	Month	Total patients with a blood culture	# Patients with single blood cultures	% Patients with single blood cultures	
2022	June	1369	239	17.5%	
2022	July	1372	248	18.1%	
2023	June	1444	236	16.3%	
2023	July	1474	249	16.9%	
2024	June	1470	239	16.3%	
**Total Pre-Intervention**		**7129**	**1211**	**17.0%**	
2024	July	754	275	36.5%	*P* < .0001

### Impact of austerity measures on positivity rate

A concern when implementing austerity measures is that there would be a deleterious impact on patient care through missed diagnoses. Ideally, austerity measures would have reduced unnecessary blood cultures which would have been negative while still allowing providers to prioritize culturing in those at greatest risk of blood stream infection. The net effect should be an increase in positivity rate. The average positivity rate, after accounting for contaminated cultures, for the academic 2022 and 2023 years was 8.8%. In July of 2022 and 2023 specifically, the positivity rate was 9.8% and 10.4%, respectively. The positivity rate from July 1–21^st^, 2024 was significantly increased at 12.1% (compared to all prior months at 8.8% (*P* = < .0001)). Not only was this increased over the aforementioned average positivity rates, but it was also the highest positivity rate recorded for any individual month over the past two years (data not shown).

In the main campus ED, the average positivity rate for the 2022 and 2023 academic years was 11.1 and 10.1%, respectively (data not shown). In July of 2022 and 2023 specifically, the positivity rate was 11.8% and 11.5%, respectively. The positivity rate in the post-intervention period (July 1–21^st^, 2024) was increased at 19.5%. This was a significant increase when compared to all previous months average 10.2% (*P* < .0001), as well as the highest positivity rate for any month over the last 2 years. The next highest positivity rate in the main campus ED over the preceding two years was 15.1% in October 2022.

### Projecting the impact of moving to single set blood culture practices

To estimate the impact that a single blood culture strategy might have on patient care and missed diagnoses, an Infectious Diseases fellow (MW) analyzed all positive blood cultures in June and July of 2022 2023, and 2024. Over those periods, there were a total of 760 bacteremic patients, excluding contaminants. Of those 760 patients, 256 (33.7%) were positive in only 1 of 2 blood culture sets (Table 3).

**Table 3.** Analysis of blood cultures which were positive in 1 of 2 sets (contaminants excluded)

		Patients positive in 1 out of 2 cultures							Total		
Year	Month	S. aureus (%)	Enterobacterales (%)	Streptococcus spp. (%)	Enterococcus spp. (%)	Pseudomonas spp. (%)	Candida spp. (%)	Other (%)	Total Bacteremic Patients (All organisms)	Patients positive in 1 out of 2 cultures (N)	Patients positive in 1 out of 2 cultures (%)
**2022**	**June**	9 (6.5%)	20 (14.4%)	2 (1.4%)	5 (3.6%)	3 (2.2%)	1 (0.7%)	8 (5.8%)	138	48	34.8%
**2022**	**July**	7 (6.1%)	20 (17.5%)	1 (0.9%)	4 (3.5%)	5 (4.4%)	2 (1.8%)	4 (3.5%)	114	43	37.7%
**2023**	**June**	6 (5.4%)	21 (19.0%)	1 (1.8%)	1 (0.9%)	2 (1.8%)	3 (2.7%)	5 (4.5%)	110	40	36.4%
**2023**	**July**	7 (4.4%)	16 (10.6%)	2 (1.3%)	9 (5.7%)	3 (1.9%)	8 (5.0%)	6 (3.8%)	159	51	32.1%
**2024**	**June**	7 (4.8%)	21 (14.4%)	6 (4.1%)	3 (2.1%)	3 (2.1%)	2 (1.4%)	6 (4.1%)	145	48	33.1%
**2024**	**July 1–21**	3 (3.1%)	9 (9.6%)	3 (3.2%)	4 (4.3%)	1 (1.1%)	2 (2.1%)	4 (4.3%)	94	26	27.7%
	**Total**	39 (5.1%)	107 (14.1%)	16 (2.1%)	26 (3.4%)	17 (2.2%)	18 (2.4%)	33 (4.3%)	760	256	33.7%

Analysis of bacteremic patients with only 1 out of 2 positive cultures suggests there is some risk of regularly missing significant pathogens such as *Staphylococcus aureus, Enterococcus* spp., Enterobacterales, *Streptococcus* spp., *Candida* spp., and *Pseudomonas aeruginosa.* Less common pathogens such as *Capnocytophaga, Haemophilus* spp.*, Acinetobacter* spp., *Neisseria gonorrhoeae, Stenotrophomonas maltophilia,* and *Staphylococcus lugdunensis* were also positive in 1 out of 2 blood culture sets (Table 3).

### Changes in blood culture ordering practices

To understand how providers changed ordering practice, we looked at the number of patients who had one blood culture, versus two or more blood cultures ordered per event. In the post intervention period, providers were more likely to order a single blood culture with 36% of patients having a single blood culture ordered compared to an average of 17% in previous June and July periods (*P* < .0001) (Table 2). If we model utilization for the full month of July 2024 assuming that 17% of patients receive only a single blood culture, then VCUHS would have performed 2649 blood cultures. By comparison, in July 2022 and 2023, there were 3769 and 4159 cultures ordered, respectively (data not shown). This strongly suggests that even with the unintended shift toward single blood culture use in some patients, the institution would have still observed a reduced rate of blood culture ordering overall.

## Discussion

In response to the blood culture bottle shortage our health system implemented several measures to try and reduce usage and preserve our supply. The guidance provided across the health system was based in part on the work of Fabre et al who used diagnostic stewardship to reduce wasteful blood culturing practices.^1^ In that study they noted a 21% decrease in blood culture utilization in their medicine units. However, they did not observe a decrease in the proportion of inappropriate blood cultures which raises the concern that their guidance led to missed diagnoses. Though impossible to answer, they did observe a statistically significant increase in positivity from 8% pre-intervention to 11% post-intervention.^1^ Although our interventions were based primarily on the work of Fabre et al, several other references were used to inform guideline development.^4–7^

Although there were significant differences between VCUHS interventions and those of Fabre et al, VCUHS observed comparable results. We relied heavily on e-mail and electronic communication as well as best practice alerts in the electronic medical record, in contrast to Fabre et al. who also utilized in-person education. Despite differences in delivery, the content of our guidance was similar to that of Fabre et al. Across the health system, we observed a 35.6% decrease in blood culture utilization in the post-intervention period (*P* < .0001) (Table 1). Especially impactful was the greater than 40% reduction in main campus, CMH, and Tapp EDs.

Pre- and post-intervention blood culture positivity rates were measured to assess whether austerity interventions were detrimental to patient care. We hypothesized that if providers had effectively reduced blood culture orders in patients who were at low-risk of bacteremia (ie cultures likely to be negative), the overall positivity rate would increase. Indeed, we saw a system-wide increase from 8.8% to 12.1% (*P* = .0115), nearly identical to the 3% increase observed by Fabre et al (*P* < .001). Further, we saw an increase from 10.2% to 19.5% (*P* < .0001) in the main campus ED, the highest blood culture volume ED at VCUHS. This suggests that providers are effectively prioritizing high-risk patients for blood culturing. In addition, we compared the absolute number of bacteremic patients diagnosed pre- and post-intervention. In July of 2022 and 2023, VCUHS diagnosed 114 and 159 individual patients with bacteremia compared to 94 in the post-intervention period (July 1–21, 2024). Projected over the entire month of July 2024, 125 patients would be diagnosed with bacteremia under post-intervention conditions, roughly equal to the average of July 2022 and 2023 (136.5 bacteremic patients/month). This further supports the conclusion that austerity measures have not contributed to a large number of missed bacteremias.

There is strong evidence that at least 40 mls of blood is required to maximize the sensitivity of a blood culture event and lower volume blood cultures risk falsely negative results.^8^ Each BACTEC bottle (both aerobic and anaerobic) can take a maximum of 10 mls. The literature would suggest nearly 20% of bacteremic patients could be missed by moving to a 1 blood culture set strategy (20 mls).^8^ To evaluate the potential impact of a single blood culture strategy, we assessed all bacteremic patients diagnosed in June and July of 2022 2023, and 2024 and determined how many of those patients were positive in 1 out of 2 blood culture sets (excluding contaminated cultures). As a means of estimating the risk of a falsely negative blood culture, we assume that there is a 50% chance that the diagnosis would have been missed in patients who were positive in only 1 out of 2 cultures. Table 3 shows that 760 total patients were bacteremic in those months with 256 (33.7%) being positive in only 1 out of 2 sets. Based on the logic outlined above, 128 (17%) of blood cultures might be missed by collecting only 1 blood culture per event. For this reason, we continued to recommend two blood cultures per event throughout the blood culture bottle shortage.

Unfortunately, in analyzing the practices of the health system, providers shifted to ordering single blood cultures with greater frequency than they had previously. In June and July of 2022 and 2023, and June of 2024, an average of 85.3% of patients had 2 blood cultures ordered per event. During the austerity period, that number decreased to 64%, suggesting that some providers adopted the mentality of conserving blood cultures by shifting to a single blood culture strategy. This shift does not account for the totality of decreased ordering but appears to be a contributing factor.

There are some limitations to these analyses. First, historical data was used as a comparator to the post-intervention period, and it is possible that random fluctuations in blood culture usage and positivity may have contributed to the differences we observed. To mitigate this possibility, we utilized comparator data from June and July of 2022 and 2023, and June of 2024 with the rationale that as an academic medical center in the mid-Atlantic, these periods should represent consistent practice and epidemiology of bacteremia. Of note, although our austerity interventions were introduced on June 24th and 27th, we used a period of July 1–21st to analyze changes in positivity rate. This was done to allow messaging to disseminate, but also so that positive cultures collected before the 24^th^ would not be counted in the post-intervention period. Another limitation is the short post-intervention period. It is possible that the changes in practice observed will not be maintained over the duration of the crisis. More data would certainly strengthen our findings, however, given the urgency of this crisis and its far-reaching impact, we thought it important to publish this dataset so that others might benefit from what we have learned and deploy these measures in their own institutions.

In summary, early evidence suggests that these measures can be implemented with minimal impact on patient care. Positivity rates have increased, and the absolute number of patients diagnosed with bacteremia is predicted to be unchanged, despite significant reductions in usage. Given these findings we believe these austerity recommendations should become the new normal and suggest there has been significant waste in blood culture utilization which can be eliminated without any obvious detriment to patient care. Moving forward, we plan to retain the decision support which is embedded in the blood culture order. In addition, we plan to revise our blood culture usage education to include all of the guidance that was put into place during this crisis. We remain concerned that providers have shifted towards ordering a single blood culture to diagnose bacteremia. We will be addressing this in our educational efforts and explore electronic mechanisms by which we can encourage the ordering of paired blood cultures.
